# The Beneficial Effects of Melatonin Administration Following Hypoxia-Ischemia in Preterm Fetal Sheep

**DOI:** 10.3389/fncel.2017.00296

**Published:** 2017-09-22

**Authors:** Tamara Yawno, Mawin Mahen, Jingang Li, Michael C. Fahey, Graham Jenkin, Suzanne L. Miller

**Affiliations:** ^1^The Ritchie Centre, Hudson Institute of Medical Research, Clayton VIC, Australia; ^2^Department of Obstetrics and Gynaecology, Monash University, Clayton VIC, Australia; ^3^Department of Paediatrics, Monash Medical Centre, Clayton VIC, Australia

**Keywords:** melatonin, white matter injury, oligodendrocytes, preterm, fetal sheep, umbilical cord occlusion, hypoxia-ischemia, oxidative stress

## Abstract

Melatonin (MLT) is an endogenous hormone that controls circadian cycle. MLT has additional important properties that make it appealing as a neuroprotective agent—it is a potent anti-oxidant, with anti-apoptotic and anti-inflammatory properties. MLT is safe for administration during pregnancy or to the newborn after birth, and can reduce white matter brain injury under conditions of chronic fetal hypoxia. Accordingly, in the current study, we examined whether an intermediate dose of MLT could restore white matter brain development when administered *after* an acute hypoxic ischemic (HI) insult in preterm fetal sheep. Fifteen fetal sheep at 95–98 days gestation were instrumented with femoral artery and vein catheters, and a silastic cuff placed around the umbilical cord. At 102 days gestation, the cuff was inflated, causing complete umbilical cord occlusion for 25 min in 10 fetuses, to induce acute severe HI. Five HI fetuses received intravenous MLT for 24 h beginning at 2 h after HI. The remaining five fetuses were administered saline alone. Ten days after HI, the fetal brain was collected from each animal and white and gray matter neuropathology assessed. HI caused a significant increase in apoptotic cell death (TUNEL+), activated microglia (Iba-1+), and oxidative stress (8-OHdG+) within the subventricular and subcortical white matter. HI reduced the total number of oligodendrocytes and CNPase+ myelin density. MLT administration following HI decreased apoptosis, inflammation and oxidative stress within the white matter. MLT had intermediate benefits for the developing white matter: it increased oligodendrocyte cell number within the periventricular white matter only, and improved CNPase+ myelin density within the subcortical but not the striatal white matter. MLT administration following HI was also associated with improved neuronal survival within the cortex. Neuropathology in preterm infants is complex and mediated by multiple mechanisms, including inflammation, oxidative stress and apoptotic pathways. Treatment with MLT presents a safe approach to neuroprotective therapy in preterm infants but appears to have brain region-specific benefits within the white matter.

## Introduction

The survival of preterm babies has improved over recent decades, but remains associated with increased rates of preterm birth-related disorders, such as retinopathy, bronchopulmonary dysplasia, necrotizing enterocolitis, and brain developmental disorders (Wen et al., 2004). Brain injury and neurodevelopmental disabilities resulting from preterm birth, which may be mediated via a hypoxic ischemic (HI) event, are a major public health concern. Preterm birth survivors have a high risk of long-term clinical, educational, and social problems; 10–15% of very preterm infants (born <32 weeks gestation) who survive will develop cerebral palsy and more than 40% will have associated motor and cognitive deficiencies at 8 years of age (Marret et al., 2013). Even those infants born moderate to late preterm (32–34 weeks gestation) will have a high risk of developmental delay compared to infants born at term (Cheong et al., 2017). Recently, Millar et al. (2017) reported the clinical impact of neonatal HI around the globe, indicating that it accounts for 23% of infant mortality worldwide, and affecting 0.7–1.2 million infants annually. In developed countries, the incidence has not decreased in the past two decades, remaining the major cause of mortality and disability.

Injury to the white matter of the developing brain, particularly the white matter surrounding the ventricles (termed periventricular leukomalacia; PVL), is the most recognized form of brain injury in preterm infants (Volpe, 2001) and can lead to severe neurological deficits (Al Rifai and Al Tawil, 2015). Although the etiology of PVL in preterm infants remains uncertain, cerebral ischemia and inflammation during brain development are predominant causes (Rezaie and Dean, 2002), resulting in neuropathology via microglial activation, excitotoxicity, and free radical attack (Volpe, 2001). Currently there are no treatments specifically designed to prevent preterm brain injury and PVL. Hypothermia is the only current therapy for term infants following severe HI; however, this treatment is considered inappropriate for preterm infants (Gancia and Pomero, 2011; Rees et al., 2011). Consequently, new therapies are required.

A strong body of evidence now supports that melatonin (MLT) is neuroprotective for acute hypoxic-ischemic perinatal brain injury, mediated via its anti-oxidant, anti-apoptotic, and anti-inflammatory properties (Hassell et al., 2015). MLT is safe for administration during pregnancy or to the newborn after birth, and can reduce white matter brain injury under conditions of chronic fetal hypoxia (Miller et al., 2014). In small and large animal models of acute perinatal HI, MLT treatment reduces apoptotic neuronal loss and markers of oxidative stress, such as hydroxyl radical production and lipid peroxidation products, and ameliorates secondary energy failure within the brain (Wakatsuki et al., 2001; Watanabe et al., 2004; Miller et al., 2005; Yawno et al., 2012; Robertson et al., 2013). MLT also possesses immune-modulating properties to promote neuroprotection following HI (Hutton et al., 2009; Balduini et al., 2012; Robertson et al., 2013).

To date, research has predominantly focused on the neuroprotective effects of MLT for HI encephalopathy in term infants, with the exception of two studies in preterm fetal sheep (Welin et al., 2007; Drury et al., 2014), where MLT was administered prophylactically or very soon after the insult. Presently, no neuroprotective agents are administered to extremely preterm or very preterm infants after birth, despite these infants being at risk for neonatal encephalopathy and long-term neurological deficits (Gopagondanahalli et al., 2016). MLT is an excellent candidate for treatment in this group, as it is essential for normal fetal neurodevelopment (Hassell et al., 2015), but is undetectable following preterm birth (Merchant et al., 2013).

In this study, we aimed to replicate the potential use of MLT therapy for preterm infants after a HI event at birth, by investigating the administration of a therapeutic (intermediate) dose of MLT commencing 2 h after HI insult and continuing for 24 h. We studied the neuroprotective effects of MLT at ∼100 days of fetal sheep gestation, approximating brain development of the human preterm infant born at 28–30 weeks gestation (Back et al., 2006). We induced HI in preterm fetal sheep by umbilical cord occlusion, and at 10 days after HI, we examined whether MLT demonstrated neuroprotective properties for the developing white and gray matter. We hypothesized that MLT given after HI to the preterm fetus would protect the developing brain.

## Materials and Methods

### Animals and Surgical Preparation

All surgical and experimental procedures were approved by the Monash Health Animal Ethics Committee (MMCA/2013/17). Pregnant Merino-Border Leicester ewes of known gestational age (carrying singleton or twin fetuses as indicated below) were used for this study. The ewes were kept in individual cages with free access to food and water under a 12-h light/dark cycle (lights on, 07:00 h).

At 95–98 days of fetal gestation (term is ∼147 days), surgery was performed on the ewe under isoflurane (Isoflo; Abbott, Sydney, NSW, Australia) general anesthesia for implantation of polyvinyl catheters to the fetus (Dural Plastics, Silverwater, NSW, Australia). Under aseptic conditions, catheters were inserted into a fetal femoral artery, femoral vein, and amniotic cavity as described by us previously (Yawno et al., 2017). An inflatable silastic cuff (16HD, In Vivo Medical, United States) was placed around the umbilical cord. This cuff could later be inflated with sterile water to cause complete cord occlusion. The fetus was returned to the uterus, and catheters were exteriorized through an incision in the ewe’s flank. A maternal jugular vein catheter was also inserted at surgery, via which antibiotics were given for 3 days post-surgery (0.1 mg/kg oxytetracycline; Engemycin MSD Animal Health, New Zealand). Antibiotics were also administered into the amniotic sac (500 mg ampicillin; Austrapen, CSL Ltd., Parkville, Australia).

### Recordings Analysis

Fetal mean arterial pressure (MAP) and heart rate (HR) were recorded throughout the experiment using pressure transducers with amplification, digitized (Power Lab, AD Instruments, Castle Hill, NSW, Australia) and recorded to a computer using Chart 5 software (AD Instruments). The MAP and HR were recorded in all fetuses for at least 24 h prior to the HI insult.

### Experimental Design

At 102.3 ± 0.2 days gestation (0.7 gestation), animals were randomized into one of three groups: (1) control (sham-occlusion + iv saline; *n* = 5); (2) HI (HI + saline; *n* = 5); (3) HI + MLT [HI + MLT; *n* = 5 (5 singles)]. HI was achieved by complete umbilical cord occlusion, in which the balloon occluder was filled with 2.0–2.5 ml sterile water for 23–25 min. The occlusion was discontinued at 25 min or sooner if the occlusion was >23 min duration and MAP had decreased to <8 mmHg. Fetal arterial blood samples (approximately 1.5 ml) were collected 24 h before, during, and 4, 6, 12, 24, 48, 72, 120, and 240 h after HI for blood gas measures (ABL 700, Radiometer, Copenhagen, Denmark) and MLT and malondialdehyde (MDA) concentrations. Plasma samples were stored at -80°C until assayed.

MLT was prepared by dissolving the bolus dose in 25 μl of 70% ETOH and the maintenance dose in 300 μl of 70% ETOH and delivered with 2.5 and 48 ml of saline, respectively. MLT treatment commenced at 2 h after the HI ceased, with fetuses receiving 0.2 mg MLT bolus i.v., followed by 0.1 mg/h i.v. for the next 24 h. MLT concentration was assessed in fetal plasma samples.

The ewe and fetus were euthanazed 10 days after HI (112.5 ± 0.2 days gestation) with a maternal i.v. overdose of pentobarbital (Lethabarb Virbac Pty Ltd., Peakhurst, Australia). The fetal brain was immediately removed from the skull, weighed and cut in half sagittally. The right hemisphere was placed in a custom-made mold, shaped to fit the fetal sheep brain, and coronal sections were obtained by cutting through the hemisphere at 0.5 cm intervals. These slices were fixed by immersion in 10% formalin for 5 days, prior to embedding in paraffin. Subsequently, 10 μm sections were cut for histological analysis.

### Melatonin Assay

Fetal plasma MLT concentrations were assayed using a commercial kit (RK-MEL2; Bühlmann Laboratories AG, Switzerland), following the manufacturer’s instructions. Briefly, MLT was extracted from the plasma using C18 reverse phase extraction columns, then assayed by a double-bind radioimmunoassay using the Kennaway G280 anti-MLT antibody raised in goat (Vaughan, 1993). MLT concentration in samples was measured against a standard curve based on the percentage bound of [125I]-2-iodomelatonin. The assay sensitivity was 1.16 pg/mL and the intra assay coefficient of variations in quality controls was 8.4%.

### Malondialdehyde Assay

Fetal oxidative stress was assessed as the concentration of lipid peroxidation in fetal plasma via the thiobarbituric acid reactive substances method used to measure MDA. The manufacturer’s protocol was followed (Cayman Chemical, Ann Arbor, United States).

### Brain Pathology

Six fields of view over two duplicate slides per brain region were examined and averaged for each animal. Brain areas of interest were the subventricular zone (SVZ), periventricular white matter (PVWM), subcortical white matter (SCWM), and striatum. For the cortex, the external granular layer (II) and the pyramidal layer (III) were analyzed and data combined as one cortical layer. Personnel were blinded to the experimental group during image capture and analysis.

Microglia were identified using rabbit anti-ionized calcium binding adaptor molecule 1 (Iba-1) antibody (Wako Pure Chemical Industries, Ltd., Osaka, Japan), raised against synthetic peptide corresponding to the C-terminal of Iba-1. The antibody was diluted 1:500 in phosphate-buffered saline (PBS) solution (0.1 mol/l, pH 7.4). Oligodendrocyte transcription factor 2 (Olig-2), a marker for oligodendrocytes at all stages of their lineage, was used to count total oligodendrocyte number in select areas (rabbit anti-Olig-2; 1:1000; Millipore, MA, United States). Mouse anti-CNPase (1:200; Sigma-Aldrich, St. Louis, MO, United States) was used to identify the integrity of mature myelin by measuring the density of myelinated axons. Anti-8-hydroxy-2′-deoxyguanosine (8-OHdG), was used to assess DNA damage induced by oxidative stress (1:100; JaICA, NIKKEN SEIL, Co., Ltd., Japan). A mouse anti-NeuN (1:200; Chemicon International, CA, United States) was used to identify mature neurons. All sections were treated with a secondary antibody (1:200; biotinylated anti-rabbit or anti-mouse IgG antibody; Vector Laboratories, Burlingame, CA, United States) and staining revealed using 3,3-diaminobenzidine (Pierce Biotechnology, Rockford, IL, United States). Apoptotic cell death was identified with anti-human/mouse activated caspase-3 antibody (1:1000; R&D Systems, Minneapolis, MN, United States) and the terminal deoxynucleotidyl transferase dUTP nick end labeling (TUNEL) staining procedure to detect DNA fragmentation. The manufacturer’s protocol was followed (DeadEnd Colorimetric TUNEL System, Promega Corporation, Madison, WI, United States), Using both procedures, apoptotic nuclei stained dark brown.

Double-label immunohistochemistry was carried out on two adjacent sections from three control, three HI, and three HI + MLT fetal brains by first blocking endogenous peroxidases with 0.3% hydrogen peroxidase in 50% methanol and then washing sections with sodium borohydride (10 mg/ml) in 0.1 M PBS to reduce the autofluorescence that can occur with paraffin-embedded sections. These sections were treated with a serum-free protein blocker (Dako) to prevent background staining, and incubated with either rabbit monoclonal anti-GFAP (1:400), or anti-Olig-2 (1:500) to identify astrocytes or oligodendrocytes, respectively. Sections were subsequently exposed to the appropriate mouse monoclonal antibody to identify the presence of anti-8-OHdG (1:200). Immunoreactivity was visualized with Alexa Fluor 594 goat anti-mouse (1:800; Molecular Probes, Eugene, OR, United States) and Alexa Fluor 488 goat anti-rabbit (1:800; Molecular Probes), and viewed with a fluorescent microscope (Olympus BX-41, Japan) at 400× magnification.

### Data Analysis

All assessments were conducted on coded slides or samples, with the examiner blinded to their experimental groups. Data are shown as mean ± standard error of the mean (SEM). Statistical analysis was performed with GraphPad Prism 7 (GraphPad Software, San Diego, United States). One-way analysis of variance (ANOVA) was used to compare between groups, with Tukey’s *post hoc* test when a significant difference was found. Differences were considered significant at *P* < 0.05.

## Results

### Fetal Outcomes

Twenty-five minutes of umbilical cord occlusion in the HI or HI + MLT group resulted in 16% fetal mortality rate (two fetuses), by the end of the occlusion. At 10 days after HI, fetal body and brain weights were not different at post-mortem between groups (**Table 1**).

**Table 1:** Physiological outcomes.

Variables	Control	HI	HI + MLT
Samples included in analysis, *n*	5	5	5
Female, *n* (%)	4 (80)	3 (60)	0 (0)
Twin, *n* (%)	2 (40)	1 (20)	5 (100)
HI duration, minute	0	24.2 ± 0.4	24.4 ± 0.4
**Weight**			
Brain weight, g	33.6 ± 0.5	30.8 ± 1.6	30.5 ± 1.5
Body weight, kg	1.9 ± 0.1	2.2 ± 0.2	2.2 ± 0.1
FHR, bpm			
Baseline	194.1 ± 5.6	191.3 ± 3.9	189.2 ± 6.9
End of occlusion	198.8 ± 6.0	**109.4 ± 5.0^∗#^**	**114.1 ± 13.2^∗#^**
2-4 h	192.6 ± 5.0	190.2 ± 6.5	194.0 ± 6.0
4-12 h	190.4 ± 3.6	191.5 ± 6.1	185.0 ± 6.1
12-24 h	195.2 ± 6.2	190.1 ± 8.4	174.7 ± 10.7
24-48 h	192.5 ± 4.9	198.1 ± 6.1	182.6 ± 13.8
**MAP, mm Hg**			
Baseline	34.0 ± 1.3	37.8 ± 0.9	**30.4 ± 2.0^∗^**
End of occlusion	36.2 ± 2.0	**26.6 ± 2.0^∗#^**	**20.1 ± 2.8^∗#^**
2-4 h	36.0 ± 2.4	37.8 ± 2.0	32.9 ± 3.3
4-12 h	35.8 ± 2.4	39.1 ± 0.7	33.4 ± 2.4
12-24 h	35.0 ± 2.4	39.6 ± 1.5	34.5 ± 3.5
24-48 h	35.4 ± 2.4	38.7 ± 1.9	35.0 ± 3.0
**pH**			
Baseline	7.40 ± 0.02	7.37 ± 0.01	7.35 ± 0.01
20 min	7.37 ± 0.01	**6.86 ± 0.02^∗#^**	**6.91 ± 0.06^∗#^**
12 h	7.38 ± 0.01	7.37 ± 0.01	7.38 ± 0.01
120 h	7.37 ± 0.01	7.38 ± 0.01	7.36 ± 0.03
240 h	7.38 ± 0.01	7.36 ± 0.01	7.38 ± 0.01
**PaO_2_, mm Hg**			
Baseline	24.6 ± 1.6	22.4 ± 0.6	22.8 ± 2.1
20 min	23.7 ± 0.6	**7.2 ± 1.6^∗#^**	**17.2 ± 2.9^∗#^**
12 h	23.3 ± 0.4	23.8 ± 1.2	26.6 ± 1.2
120 h	22.5 ± 0.9	25.2 ± 1.7	30.4 ± 3.5
240 h	24.6 ± 0.8	24.1 ± 2.0	27.9 ± 1.5
**PaCO_2_, mm Hg**			
Baseline	38.3 ± 3.9	46.4 ± 2.8	43.5 ± 1.9
20 min	43.8 ± 2.6	**115.0 ± 4.7^∗#^**	**90.1 ± 12.2^∗#^**
12 h	46.6 ± 2.5	46.8 ± 1.9	39.9 ± 1.9
120 h	46.5 ± 2.1	46.8 ± 2.5	39.8 ± 2.1
240 h	47.1 ± 2.2	49.0 ± 2.3	43.1 ± 2.3
**SaO_2_, %**			
Baseline	76.0 ± 1.8	69.9 ± 1.7	72.3 ± 8.0
20 min	69.2 ± 1.3	**9.1 ± 1.4^∗#^**	**20.7 ± 4.9^∗#^**
12 h	70.2 ± 1.2	72.0 ± 2.9	77.3 ± 1.9
120 h	66.3 ± 2.5	74.5 ± 2.5	77.5 ± 4.8
240 h	69.4 ± 3.1	69.7 ± 2.5	74.7 ± 2.2
**BE, mmol/l**			
Baseline	-0.3 ± 1.1	0.9 ± 1.6	-1.5 ± 0.7
20 min	1.6 ± 1.3	**-15.6 ± 1.1^∗#^**	**-16.0 ± 1.1^∗#^**
12 h	1.2 ± 1.1	1.1 ± 1.1	-1.5 ± 0.7
120 h	1.2 ± 1.1	1.8 ± 1.2	-3.7 ± 2.1
240 h	1.8 ± 1.1	1.7 ± 0.6	0.3 ± 1.0
**Glucose, mmol/l**			
Baseline	0.9 ± 0.1	0.9 ± 0.1	1.0 ± 0.1
20 min	1.0 ± 0.1	0.4 ± 0.1	0.5 ± 0.1
12 h	1.1 ± 0.1	1.5 ± 0.1	1.7 ± 0.2
120 h	1.1 ± 0.1	1.1 ± 0.1	1.2 ± 0.2
240 h	1.0 ± 0.1	1.1 ± 0.1	1.2 ± 0.1
**Lactate, mmol/l**			
Baseline	0.9 ± 0.2	0.9 ± 0.1	0.9 ± 0.1
20 min	0.8 ± 0.1	**8.6 ± 0.6^∗#^**	**6.6 ± 1.1^∗#^**
12 h	0.9 ± 0.0	1.3 ± 0.2	1.6 ± 0.3
120 h	1.0 ± 0.1	1.1 ± 0.1	1.3 ± 0.2
240 h	1.0 ± 0.1	1.5 ± 0.3	1.0 ± 0.1


*Mean (%) or mean ± SEM are presented for each group. One-way ANOVA and Tukey’s *post hoc* tests were carried out on comparisons between groups. ^∗^*P* < 0.05 vs control at the same time point. ^#^*P* < 0.05 within group comparisons vs baseline. The start of occlusion was set as time 0. HI, hypoxia ischemia; MLT, melatonin; FHR, fetal heart rate; MAP, mean arterial pressure; PaO_*2*_, partial arterial pressure of oxygen; PaCO_*2*_, partial arterial pressure of carbon dioxide; SaO_*2*_, oxygen saturation; BE, base excess. Bold values indicate significant changes.*

The HI insult caused significant hypotension and bradycardia, reduced fetal arterial pH, PaO_2_, SaO_2_, and base excess, and increased PaCO_2_ and lactate levels, compared to control levels (**Table 1**). There was no difference in the duration of HI between the HI and the HI + MLT cohorts, or any physiological parameters following the insult between groups.

**FIGURE 1:**
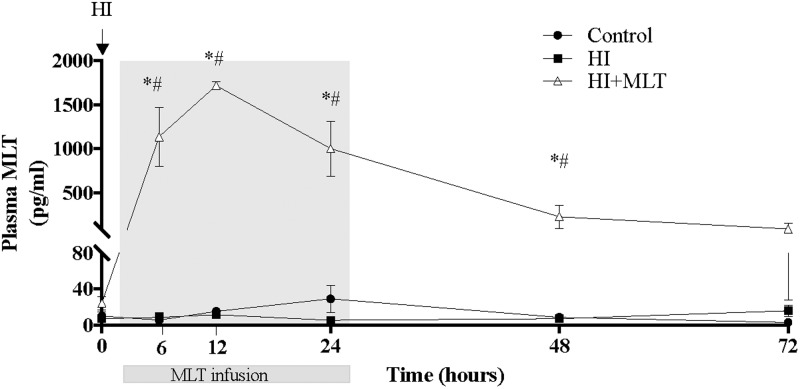
Melatonin concentration within fetal plasma in control, HI, and HI + MLT groups. The arrow indicates the start of HI; the shaded area indicates the time of melatonin infusion. Data are mean ± SEM. ^∗^Significant difference between HI group vs control group. ^#^Significant difference between treatment vs baseline values (pre-treatment). *P* < 0.05.

Circulating MLT levels were measured in all fetuses at 0, 6, 12, 24, 48, and 72 h after HI. There was no significant difference in MLT concentrations between the control group and the HI group. Compared to baseline (pre-MLT infusion values; 10.1 ± 4.4 pg/ml), MLT was significantly increased (1135 ± 337 pg/ml) at the 6 h sample (4 h after the onset of MLT infusion) in the HI + MLT group, with peak circulating MLT concentration at 10 h after HI (1717 ± 46 pg/ml, or 70-fold increase compared to baseline). MLT infusion was stopped at 26 h post-HI, however, circulating MLT continued to be significantly elevated at 48 h post-HI, but returned close to baseline level by the 72 h time point (**Figure 1**).

### Plasma Malondialdehyde

**FIGURE 2:**
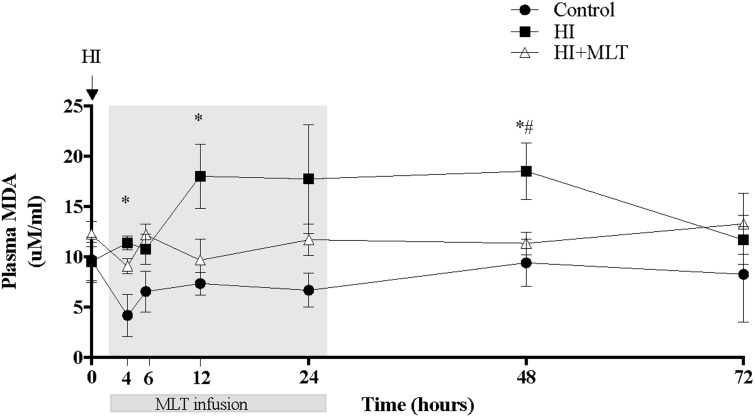
Malondialdehyde (MDA) concentration within fetal plasma in control, HI, and HI + MLT groups. MDA increased in HI compared to control and HI + MLT. The arrow indicates the start of HI, the shaded area indicates the time of melatonin infusion. Data are mean ± SEM. ^∗^Significant difference between HI group vs control group. ^#^Significant difference between treatment vs baseline values (pre-treatment). *P* < 0.05.

There was no change in circulating fetal MDA concentrations in control fetuses over the duration of the study (**Figure 2**). Compared to control animals, HI was associated with an increase of fetal plasma MDA levels from 4 h post-HI through to 48 h, with a return to baseline at 72 h. When HI fetuses were treated with MLT from 2 h post-HI (HI + MLT cohort), there was no significant increase in MDA levels at any time point; and MDA concentration was significantly reduced at 48 h compared to HI fetuses (**Figure 2**).

### Brain Pathology

**FIGURE 3:**
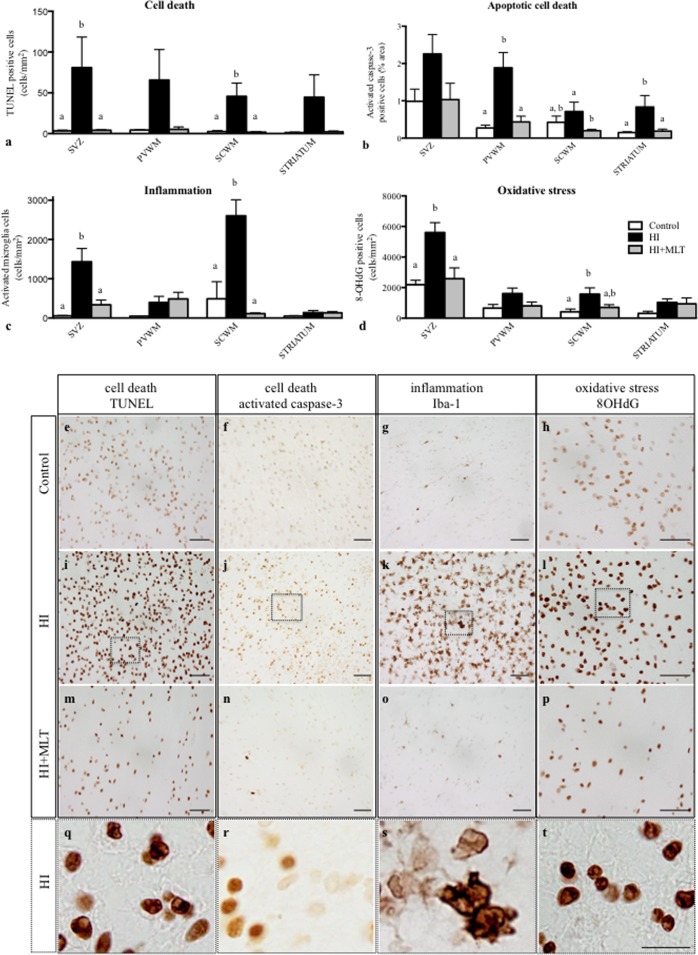
The number of TUNEL positive cells **(a)**, activated caspase-3 positive cells **(b)**, activated microglia cells **(c)**, 8-OHdG positive cells **(d)** in the subventricular zone (SVZ), periventricular white matter (PVWM), subcortical white matter (SCWM), and striatum in the fetal brain 10 days after sham treatment (*n* = 5), HI (*n* = 5) and HI + MLT (*n* = 5). Comparisons are made within the same brain region and not across regions. Each bar represents the mean ± SEM; values that do not share a common letter are significantly different from each other, *P* < 0.05. Representative photomicrographs showing TUNEL **(e,i,m)**, activated caspase-3 **(f,j,n)**, Iba-1 **(G,K,O)**, and 8-OHdG **(h,l,p)** positive staining in the SCWM in the brain of control **(e–h)**, HI **(i–l)**, and HI + MLT **(m–p)** fetuses. **(q–t)** Micrographs showing higher power magnification of **(i–l)**, respectively. Scale bars = 50 μm **(e–p)** and 12.5 μm **(q–t)** as shown in **(t)**.

In HI fetuses, we observed an increase in activated caspase-3 and TUNEL-positive cells within all brain regions examined (**Figures 3a,b**); TUNEL-positive cell counts were significantly increased in the SVZ and SCWM (81.0 ± 37.5 and 45.7 ± 16.2 cell/mm^2^) compared to control fetuses (3.3 ± 1.0 and 2.4 ± 1.3 cell/mm^2^) and activated caspase-3 cells were significantly elevated in the PVWM and striatum (1.9 ± 0.4 and 0.8 ± 0.3%) compared to control fetuses (0.3 ± 0.07 and 0.15 ± 0.02%). MLT treatment ameliorated apoptotic cell death within all brain regions examined; TUNEL-positive cells were returned to control levels and were significantly reduced in the SVZ and SCWM (4.1 ± 0.7 and 1.7 ± 1.0 cell/mm^2^; **Figure 3a**) compared to HI alone (**Figures 3e,i,m,q**). Similarly, activated caspase-3 positive cells were returned to control levels and were significantly reduced in the PVWM, SCWM, and the striatum (0.4 ± 0.2, 0.2 ± 0.04, and 0.2 ± 0.05%) in the HI + MLT fetuses compared to HI alone (**Figures 3b,f,j,n,r**). Inflammatory cells (Iba-1 positive microglia) were observed in fetal brains of all treatment groups. Cells that stained positively for Iba-1 had the appearance of activated microglia (ameboid with large cell bodies) and resting microglia (ramified, with small cell bodies and long branching processes) as described previously (Yawno et al., 2013). In HI fetuses, there was a significant increase in the number of activated microglia in the SVZ (1431 ± 340 cell/mm^2^) and SCWM (2602 ± 408 cell/mm^2^; **Figure 3c**), when compared to control fetuses (55 ± 8.1 and 489 ± 438 cell/mm^2^, respectively). The administration of MLT significantly reduced the number of activated microglia (in SVZ 334 ± 123 cell/mm^2^ and SCWM 112 ± 19 cell/mm^2^) after HI when compared to HI alone, with no difference in the number of activated microglia between HI + MLT and control brains (**Figures 3c,g,k,o,s**). The brains of all control and HI animals expressed a degree of oxidative stress-induced DNA damage (8-OHdG positive cells). In HI fetuses, 8-OHdG positive cells were significantly elevated in the SVZ (5604 ± 644 cell/mm^2^) and SCWM (1571 ± 413 cell/mm^2^) compared to control fetuses (2188 ± 296 and 414 ± 179 cell/mm^2^, respectively; **Figure 3d**). The administration of MLT significantly reduced oxidative stress-induced DNA damage (in SVZ 2584 ± 705 cell/mm^2^ and SCWM 407 ± 177 cell/mm^2^), with no difference in the number of 8-OHdG cells between HI + MLT and control brains (**Figures 3d,h,l,p,t**).

**FIGURE 4:**
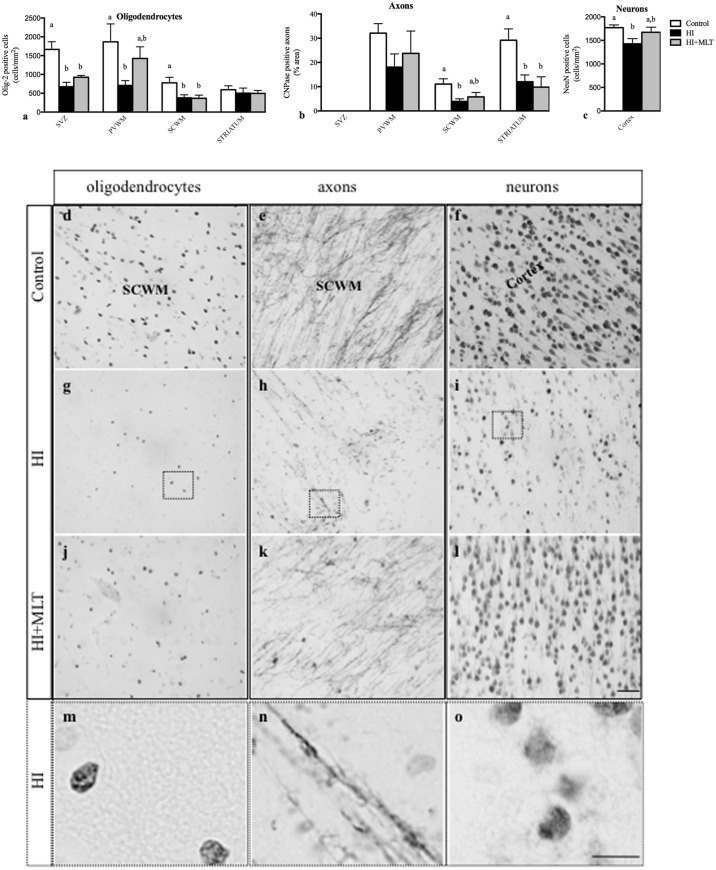
The number of Olig-2 positive cells **(a)**, and the expression of CNPase positive axons as a percentage area **(b)** in the subventricular zone (SVZ), periventricular white matter (PVWM), subcortical white matter (SCWM), and striatum; and the number of NeuN positive neurons **(c)** within the cortex in the fetal brain 10 days after sham treatment (*n* = 5), HI (*n* = 5), and HI + MLT (*n* = 5). Comparisons are made within the same brain region and not across regions. Each bar represents the mean ± SEM; values that do not share a common letter are significantly different from each other *P* < 0.05. Representative photomicrographs showing Olig-2 **(d,g,j)**, CNPase **(e,h,k)**, and NeuN **(f,i,l)** positive staining in the SCWM in the brain of control **(d–f)**, HI **(g–i)**, and HI + MLT **(j–l)** fetuses. **(m–o)** Micrographs showing higher power magnification of **(g–i)**, respectively. Scale bars = 50 μm (day l) as shown in l and 12.5 μm **(m–o)** as shown in **(o)**.

Olig-2 positive oligodendrocytes were prevalent in the white matter of all fetal brains examined. The number of Olig-2 positive cells was significantly reduced in the SVZ, PVWM, and SCWM of HI fetuses (674 ± 118, 710 ± 127, 380 ± 81 cell/mm^2^, respectively) compared to control fetuses (1666 ± 204, 1868 ± 475, 777 ± 148 cell/mm^2^, respectively; **Figure 4a**). MLT administration following HI ameliorated the decrease of Olig-2 positive cell within the PVWM (1426 ± 307 cell/mm^2^) but not in the SVZ and SCWM (**Figures 4a,d,g,j,m**). CNPase immunostaining revealed that myelin density was significantly reduced in the HI group compared to control. In control brains, white matter tracts had well-organized, densely distributed myelinated fibers but, in contrast, the white matter of HI brains appeared fragmented and disorganized, with significantly reduced myelin density within the SCWM (3.9 ± 1.1%) and the striatum (12.1 ± 2.8%; **Figure 4b**). HI + MLT fetuses demonstrated restored myelin morphology that was well organized with dense myelin within the SCWM (5.9 ± 1.8%) but was not improved in the striatum (**Figures 4b,e,h,k,n**). The number of NeuN positive neurons was significantly reduced within the cortex of HI fetuses (1427 ± 110 cell/mm^2^) compared to control fetuses (1771 ± 58 cell/mm^2^). MLT administration following HI ameliorated the decrease of NeuN positive cells (1673 ± 108 cell/mm^2^; **Figures 4c,f,i,l,o**).

**FIGURE 5:**
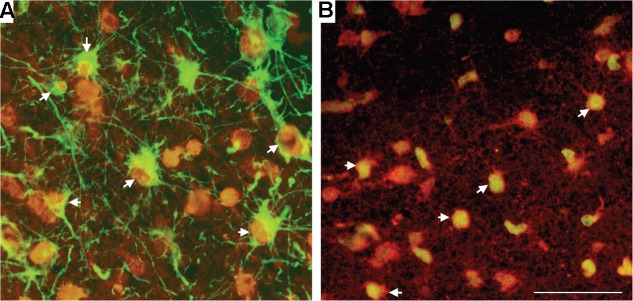
Double-label immunohistochemistry in the SCWM for 8-OHdG, together with astrocytes (GFAP) **(A)** and oligodendrocytes (Olig-2) **(B)** in a HI brain. Cell types (GFAP and Olig-2) were visualized with Alexa Fluor 488 (green) and 8-OHdG with Alexa Fluor 594 (red). White arrowheads indicate cells bodies that are co-localized for the cell marker and for 8-OHdG. Scale bar = 50 μm as shown in **(B)**.

Within the SCWM, we used double-label immunohistochemistry to demonstrate that it was predominantly astrocytes and oligodendrocytes that were susceptible to oxidative stress-induced DNA damage following HI. Approximately 50% of 8-OHdG-positive cells were positive for GFAP (astrocytes; **Figure 5A**), with the remaining 50% of SCWM cells that stained positive for 8-OHdG also being positive for Olig-2 (oligodendrocytes; **Figure 5B**).

## Discussion

This study examined the neuroprotective benefits of MLT administered according to a clinically relevant protocol in response to preterm HI brain injury. We administered MLT from 2 h after HI insult and assessed brain development and brain injury after 10 days, at ∼100 days of fetal sheep gestation, targeting human brain development equivalent to an infant born at approximately 28–30 weeks (Back et al., 2006). We show that MLT reduces circulating markers of oxidative stress for the period of MLT administration (24 h) and for up to 24 h after MLT treatment was stopped. This sustained anti-oxidant effect of MLT corresponded to the period of sustained elevation in circulating MLT concentration above baseline levels.

HI caused significant neuropathology, characterized by cell death, neuroinflammation, oxidative stress-induced DNA damage, neuronal loss, and white matter injury. MLT administration prevented neuronal loss and was partially protective of the developing white matter. MLT ameliorated oligodendrocyte cell death within the PVWM, but not subcortical or subventricular areas, and MLT did not normalize CNPase-positive myelin density within the white matter areas examined. We show that the neuroprotective benefits of MLT were mediated by its anti-apoptotic, anti-inflammatory, and anti-oxidant actions within the preterm brain.

There are several neuroprotective agents investigated as treatment for perinatal brain injury. When compared in a systematic manner for key criteria that include *ease of administration*, *adverse effects*, and *overall benefit and efficacy*, MLT was ranked the highest as a potential rescue therapy for neonatal encephalopathy (Robertson et al., 2012). The optimal neuroprotective dose or circulating concentration of MLT is not yet known, but, to date, most studies to assess MLT as a neuroprotective agent for perinatal brain injury demonstrate significant benefits with the use of high concentrations (≥10 mg/kg to the mother or offspring; as reviewed in Hassell et al., 2015). In addition, most animal studies to date have examined the effects of MLT on brain injury induced at term or close to term, reflecting administration for term HI encephalopathy (reviewed in Hassell et al., 2015).

In the current study, we examined MLT administration following preterm HI brain injury, using an intermediate dose of MLT comprising a 0.2 mg bolus i.v. to the fetus at 2 h after HI followed by an infusion of 0.1 mg/h for 24 h. This dosing resulted in a 70-fold increase in circulating MLT concentration in the fetus, which peaked mid infusion at 12 h and remained sustained for 24 h following MLT infusion. This represents slow MLT clearance in the preterm fetus, consistent with a longer MLT half-life in preterm infants compared to healthy term newborns or adults (Merchant et al., 2013). We conclude that, as a result, clinically it may not be necessary to administer MLT continuously. Further, in this study we demonstrate that following preterm HI, MLT acts as an anti-oxidant with anti-apoptotic, and anti-inflammatory properties within the developing brain. In turn, MLT is partially protective for oligodendrocytes and assists myelination. Our results are supported by two previous studies in preterm fetal sheep in which MLT showed robust cerebral anti-oxidant and anti-inflammatory properties, and decreased cell death, but was only partially neuroprotective for neuronal survival (Welin et al., 2007; Drury et al., 2014). Interestingly, in the current study and that of Drury et al. (2014), the protective effects of MLT within the white matter were region specific; both studies reporting that oligodendrocyte cell number (either Olig-2+ or CNPase+) was preserved in the PVWM but not in other white matter regions examined. This region specific effect is yet to be determined, but it may be due to changes in blood flow to different regions of the white matter. Hypoxia can significantly shift the frequency of smaller to larger blood vessels in periventricular and SCWM but not the gray matter, resulting in altered cerebral blood flow and increased brain injury (Baburamani et al., 2013). MLT has been shown to decrease cerebral blood flow in young rats (Capsoni et al., 1995) and differentially affects vascular blood flow in humans (Cook et al., 2011), which might explain why MLT has preferentially preserved the PVWM. However, closer examination of the frequency and distribution of blood vessel size within the white matter regions in this study are ongoing.

Plasma MDA levels were significantly elevated 4 h post-HI and remained high for 48 h, with this oxidative stress response being prevented by MLT administration. This finding complements the work performed in fetal sheep and human infants that showed elevated MDA concentration up to four times higher than baseline after HI (Fulia et al., 2001; Fraser et al., 2008). MLT administration reduces MDA concentration in hypoxic newborn rats and term infants following perinatal asphyxia because of its anti-oxidant capacities (Fulia et al., 2001). These anti-oxidant properties of MLT are vital for protecting the developing fetal brain since oxidative stress is a critical mediator of brain injury and functional deficits. Thus reducing this adverse response following acute hypoxia may protect against cellular damage. In our study HI caused a significant increase in oxidative stress within the white matter of the brain, and the administration of MLT in HI fetuses had a strong anti-oxidant effect. It is widely considered that the protective effects of MLT are principally due to its anti-oxidant effects and our results support the anti-oxidant capacity of MLT within the immature brain, but it is also known that MLT has other actions that mediate neuroprotection, including anti-inflammatory effects. Indeed, oxidative stress and inflammation are intimately linked (Miller et al., 2012); reactive oxygen species upregulate inflammatory cytokine production via nuclear factor-κB activation (Li and Karin, 1999) and conversely, activation of brain microglia induces the release of free radicals (Dringen, 2005). We did not set out to separate the anti-oxidant and anti-inflammatory effects of MLT, but the fact that MLT can mediate both of these injury-inducing pathways in the developing brain is certainly beneficial in the setting of preterm HI brain injury. We have shown previously that acute HI in fetal sheep induces a biphasic increase in hydroxyl radical release within the brain, and that MLT is able to ameliorate increase of this highly damaging reactive oxygen species via receptor and non-receptor-mediated mechanisms (Miller et al., 2005). In the clinical situation, the anti-inflammatory effects of MLT could be highly important in infants born preterm and requiring ventilation, given the known link between mechanical ventilation and systemic and cerebral inflammation, which may lead to brain injury (Barton et al., 2016). Other potential neuroprotective mechanisms of MLT have been examined, and reviewed (Hassell et al., 2015), and in the setting of preterm HI insult, are likely to include stabilization of the blood–brain barrier (Yawno et al., 2017) and reducing neuronal death secondary to glutamate excitotoxicity (Buendia et al., 2015).

Ten days after HI, fetal neuropathology was evident. We found region-specific white matter injury within the developing brain, with MLT treatment mediating a preferential protection to the PVWM only (reflecting oligodendrocyte injury). This is an important observation given the critical link between periventricular injury (such as PVL) and long-term neurological deficits (Back, 2006). Olivier et al. (2009) induced chronic hypoxia in developing rats and showed that MLT was able to normalize oligodendrocyte development, principally via actions to decrease microglial activation. Similarly in our study we observed that MLT moderated the neuroinflammatory response to acute insult within the SVZ and SCWM, however, oligodendrocytes were not protected by MLT in these two brain regions. This observation is indicative that the anti-inflammatory actions of MLT alone do not protect white matter brain development.

Apoptotic cell death was initiated in the SVZ, PVWM, SCWM, and striatum following 25 min HI, with elevated TUNEL+ DNA fragmentation and activated caspase-3, evident at 10 days after the HI insult. This delayed apoptosis is mediated partly through the upregulation of pro-apoptotic proteins such as the caspases, with some processes lasting days to weeks (Dell’Anna et al., 1997). MLT treatment reduced both markers of apoptotic cell death in this study, consistent with previous findings (Welin et al., 2007; Yawno et al., 2012; Drury et al., 2014). This action of MLT is mediated by the downregulation of pro-apoptotic protein caspase-3 as seen in this study and an upregulation of anti-apoptotic proteins (Yang et al., 2015). The anti-apoptotic properties of MLT are likely a key neuroprotective benefit of MLT treatment and, indeed, we found that MLT significantly decreased the HI-induced neuronal cell loss within the cortex.

We further investigated the pathology within the white matter and found that oligodendrocyte cell numbers (Olig-2 positive cells) were significantly reduced in HI animals. Olig-2 staining accounts for all oligodendrocytes, including the pre-, immature, and mature myelinating oligodendrocytes (Back et al., 2012). We performed double-label staining to investigate whether the oligodendrocytes were vulnerable to oxidative stress-induced DNA damage (Olig-2 and 8-OHdG), indeed, we showed that ∼50% of cells demonstrating oxidative stress were oligodendrocytes. We further showed that within white matter brain regions, it was the astrocytes that were also vulnerable to oxidative stress. MLT administration following HI was protective to these cells within the SVZ and SCWM. This is an important finding since astrocyte injury has been associated with mediating toxic edema, provoking inflammation, releasing cytotoxins, and forming scars that inhibit axonal regeneration (Sofroniew, 2005). In the current study, we do not show quantitative data on GFAP positive astrocytes; however, we have recently shown that maternal MLT treatment reduced GFAP-positive astrogliosis in the fetal brain after HI, and restored normal morphology of astrocytes (Yawno et al., 2012). Previous *in vitro* studies have also shown that MLT can protect astrocytes against excitotoxicity and oxidative stress following CNS injuries (Das et al., 2008). MLT significantly reduces the loss of oligodendrocytes, demyelination, and axonal injury in models of hypoxia, stroke, and multiple sclerosis (Kaur et al., 2010; Villapol et al., 2011; Wen et al., 2016). MLT was also able to increase oligodendrocyte differentiation *in vitro* (Ghareghani et al., 2016), supporting its therapeutic potential following oligodendrocyte and myelin pathologies. In the current study, we did not examine protein and mRNA levels for markers of oxidative stress and brain injury, and we acknowledge that this would be useful in future studies to unravel the specific mechanisms of action by which MLT mediates protective benefits. Our results do, however, support that MLT has specific neuroprotective effects at the cellular level, and these may be region specific, but are likely to be predominantly mediated by an anti-oxidant action of MLT.

This is the first study to delay MLT administration to 2 h after an acute perinatal insult, and to assess resulting neuropathology in the preterm brain. Our rationale for delaying administration and giving an intermediate dose of MLT was to mimic the clinical situation when high-risk preterm infant are born and treatment may not commence immediately. In this scenario, MLT was beneficial to the preterm brain by decreasing cellular apoptosis, inflammation and oxidative stress. A potential limitation of the study is that we administered saline without the vehicle (ethanol) to a control group. The Drury et al.’s (2014) study showed that ethanol had specific effects, but the Welin et al.’s (2007) study, and our study (Miller et al., 2005) did not show vehicle/ethanol effects when administered at the same dose. Despite this, it is encouraging that ethanol free MLT formulations are now being pursued (Aly et al., 2015).

The enormity of the problem of brain injury in preterm infants is due to the increased number of survival (50–70%) in recent years; the majority of these surviving infants have serious neurodevelopmental disability, including cognitive deficits and motor disability. The most common neuropathology in these premature infants is PVL (Volpe, 2009). Collectively, evidence from our animal studies, and those of others, demonstrate that inflammation, oxidative stress, and cell death play a pivotal role in the pathogenesis of PVL. Despite the region-specific benefits within the white matter, MLT represents a potential approach to reducing brain damage in preterm infants. Future studies on the length of treatment and the dosing are aspects that could be evaluated further to gain a greater understanding of how MLT might be adopted into clinical practice.

## Author Contributions

MF, GJ, and SM developed the idea, designed the experiments, and generated government funding for the project. TY helped optimize the experimental design, conducted the animal work, generated and analyzed data, and wrote the manuscript. MM and JL provided intellectual and experimental input, and helped with the animal work and generated data. All the authors provided feedback with the preparation of the manuscript.

## Conflict of Interest Statement

The authors declare that the research was conducted in the absence of any commercial or financial relationships that could be construed as a potential conflict of interest.
